
JOURNAL INFORMATION
==============================
NLM Title Abbreviation: Inter Econ
Journal ID: Inter Econ
NLM Journal ID: 101086399

Inter Economics

ISSN: 0020-5346

ARTICLE INFORMATION
==============================
PMCID: PMC8490828
DOI: 10.1007/s10272-021-0992-7
Article ID: 992
Article version: 1
Subjects: Forum

The Impact of COVID-19 on Gender Equality in Europe

Electronic publication date: 2021 Oct 5
Print publication date: 2021
Volume: 56
Issue: 5
First page: 248
Last page: 248
Copyright: © The Author(s) 2021
License: Open Access: This article is distributed under the terms of the Creative Commons Attribution 4.0 International License (https://creativecommons.org/licenses/by/4.0/).
Open Access funding provided by ZBW — Leibniz Information Centre for Economics.
License URL: https://creativecommons.org/licenses/by/4.0/

issue-copyright-statement: © The Author(s) 2021

==============================

DOI: 10.1007/s10272-021-0992-7

### The Impact of COVID-19 on Gender Equality in Europe

**The COVID-19 crisis has affected working and living conditions in all EU member states, unveiling and exacerbating existing social, economic and gender inequalities. Gender differences in the labour market impacts of the pandemic pose new short-term and long-term challenges to gender equality. In the absence of targeted policies to tackle these challenges, even the progress made thus far to narrow the gender gap is at risk of being rolled back. Has the impact of the COVID-19 pandemic-driven economic crisis created a "she-cession" adversely affecting women's employment status across Europe? How has the coronavirus pandemic affected the unpaid care burden and living conditions of working parents? Has the pandemic also opened up new opportunities to correct structural gender inequalities? Which labour, family and social welfare policies and company practices could help to achieve a more gender-equal recovery? The contributions in this Forum shed light on the pandemic's direct effects on women and gender equality.**

### COVID-19 and OECD Labour Markets: What Impact on Gender Gaps?

**Monika Queisser**, Organisation for Economic Co-operation and Development, Paris, France.

### Gender Differences in the Impact of the COVID-19 Pandemic on Employment, Unpaid Work and Well-Being in the EU

**Sanna Nivakoski**, Eurofound, Dublin, Ireland.

**Massimiliano Mascherini**, Eurofound, Dublin, Ireland.

### Teleworking: A Curse or a Blessing for Gender Equality and Work-Life Balance?

**Manuela Tomei**, International Labour Organization, Geneva, Switzerland.

### Unequal Labour Market Impacts of COVID-19 in Sweden – But Not Between Women and Men

**Pamela Campa**, SITE, Stockholm School of Economics, Sweden.

**Jesper Roine**, SITE, Stockholm School of Economics, Sweden.

**Svante Strömberg**, SITE, Stockholm School of Economics, Sweden.

### Gender Equality and the COVID-19 Pandemic: Labour Market, Family Relationships and Public Policy

**Paola Profeta**, Bocconi University, Milano, Italy.
